# Harnessing Mirror Neurons: A New Frontier in Parkinson’s Disease Rehabilitation—A Scoping Review of the Literature

**DOI:** 10.3390/jcm13154539

**Published:** 2024-08-02

**Authors:** Roberto Tedeschi, Daniela Platano, Danilo Donati, Federica Giorgi

**Affiliations:** 1 Department of Biomedical and Neuromotor Sciences, Alma Mater Studiorum, University of Bologna, 40125 Bologna, Italy; daniela.platano@unibo.it; 2 Physical Medicine and Rehabilitation Unit, IRCCS Istituto Ortopedico Rizzoli, 40136 Bologna, Italy; 3 Clinical and Experimental Medicine PhD Program, University of Modena and Reggio Emilia, 41124 Modena, Italy; danilo.donati@unimore.it; 4 Physical Therapy and Rehabilitation Unit, Policlinico di Modena, 41125 Modena, Italy; 5 IRCCS Institute of Neurological Sciences, UOC Child Rehabilitation Medicine, 40138 Bologna, Italy; federica.giorgi15@gmail.com

**Keywords:** Parkinson’s disease, action observation (AO), motor imagery (MI), mirror neurons, physiotherapy

## Abstract

**Background**: Parkinson’s disease (PD) is a neurodegenerative disorder characterized by motor symptoms such as tremors, rigidity, and bradykinesia. Rehabilitation utilizing mirror neurons leverages the brain’s capacity for action observation (AO) and motor imagery (MI) to enhance motor function. This approach involves patients imitating movements observed in therapists or videos, aiming to improve gait, coordination, and overall quality of life. Mirror neuron activation facilitates motor learning and may decelerate disease progression, thus enhancing patient mobility and independence. **Methods**: This scoping review aimed to map current evidence on PD therapies employing mirror neuron-based rehabilitation. Databases searched included PubMed, PEDro, and Cochrane. The review included randomized controlled trials (RCTs) and systematic reviews that examined the effects of AO and MI in PD rehabilitation. **Results**: Five studies met the inclusion criteria, encompassing various rehabilitation techniques focusing on AO and MI. These studies consistently demonstrated positive outcomes, such as reduced disease severity and improved quality of life, gait, and balance in PD patients. The activation of mirror neurons through AO and MI was shown to facilitate motor learning and contribute to improved functional mobility. **Conclusions**: Although the included studies support the beneficial impact of AO and MI techniques in PD rehabilitation, numerous questions remain unresolved. Further research is necessary to evaluate the potential integration of these techniques into standard physiotherapy routines for PD patients. This review highlights the promise of AO and MI in enhancing motor rehabilitation for PD, suggesting the need for more comprehensive studies to validate and refine these therapeutic approaches.

## 1. Introduction

Parkinson’s disease (PD) stands as one of the most intricate and enigmatic neurological disorders known to science, affecting millions worldwide, with approximately 300,000 patients in Italy alone. First described by Dr. James Parkinson in 1817 as a progressive degeneration of the central nervous system, PD specifically targets dopamine-producing neurons located in the substantia nigra of the midbrain [1]. A hallmark of the disease is the presence of Lewy bodies—aggregates of alpha-synuclein proteins—within the neurons of the midbrain [2,3,4]. The clinical presentation of Parkinson’s disease is characterized by both motor and non-motor symptoms. Motor symptoms include resting tremors, bradykinesia, muscle rigidity, postural instability, festination, the freezing of gait (FOG), and an expressionless face (hypomimia). Non-motor symptoms encompass sleep disturbances [5], dementia [6], and pain [7,8]. These symptoms severely compromise the quality of life, independence, and autonomy of patients, imposing significant burdens on their families as well. A critical factor in assessing the quality of life in PD patients is the risk of falls. A recent systematic review reveals that fall rates among PD patients are alarmingly high, with 35% to 90% of patients experiencing at least one fall since diagnosis, and 39% of these cases being at risk of recurrence [9,10]. Despite advancements in pharmacological and conventional physical therapies, there is a growing demand for innovative and complementary rehabilitative approaches. Various therapeutic approaches have been explored, including physiotherapy with visual or auditory cues [11,12], hydrotherapy [13], tango therapy [14], and tai chi therapy [15]. These methods aim to enhance motor function, balance, and the overall quality of life by leveraging different sensory inputs and movement patterns to stimulate the nervous system and improve motor control. Among these, mirror neurons have captured the interest of researchers in neuroscience and rehabilitation. Discovered in monkey brains in 1992 and later in humans, these specialized neurons activate both when an individual performs a specific action and when they observe someone else performing it. This activation can lead to empathy in actions and plays a crucial role in motor learning processes [16]. Action observation (AO) and motor imagery (MI) are cognitive processes that activate mirror neurons, allowing individuals to form a mental image of an observed gesture, visualize it mentally, and subsequently reproduce it. This innovative approach leverages the brain’s ability to learn by observing others’ actions and mentally performing visualized actions, thus offering a promising avenue for enhancing motor function, gait coordination, and the overall quality of life for patients with Parkinson’s disease. The activation of mirror neurons enhances motor learning and potentially slows disease progression, thus improving patients’ mobility and independence. Although conventional Parkinson’s disease therapies have advanced significantly, incorporating novel rehabilitative techniques [17,18] that utilize mirror neurons could further enhance patient outcomes. These innovative methods underscore the necessity for ongoing research and the integration of multifaceted therapeutic strategies to effectively address the complexities of Parkinson’s disease [19].

Rehabilitation involving mirror neurons typically includes several key steps:

1. Action Observation (AO): Patients watch videos or live demonstrations of specific movements or tasks. These can include activities such as walking, reaching, or balancing exercises. The goal is to engage the mirror neuron system by closely observing the actions [20].
2. Motor Imagery (MI): After observing the action, patients are guided to mentally rehearse the observed movements without physically performing them. This mental practice helps to reinforce the neural pathways involved in the action [21].
3. Physical Execution: Patients then physically attempt to perform the observed and mentally rehearsed actions. This step helps to consolidate the motor learning that has been initiated through observation and imagery.
4. Feedback and Adjustment: During the physical execution of the movements, patients receive feedback from therapists to correct and refine their movements. This iterative process ensures that the motor learning is accurate and effective.
5. Integration into Daily Activities: The learned movements and exercises are gradually integrated into the patients’ daily routines to enhance functional mobility and independence.

Action observation (AO) and motor imagery (MI) are cognitive processes that activate mirror neurons, allowing individuals to form a mental image of an observed gesture, visualize it mentally, and subsequently reproduce it [19,22]. This innovative approach leverages the brain’s ability to learn by observing others’ actions and mentally performing visualized actions, thus offering a promising avenue for enhancing motor function, gait, coordination, and the overall quality of life for patients with Parkinson’s disease. The activation of mirror neurons enhances motor learning and potentially slows disease progression, thus improving patients’ mobility and independence. Although conventional Parkinson’s disease therapies have advanced significantly, incorporating novel rehabilitative techniques that utilize mirror neurons could further enhance patient outcomes [22,23]. These innovative methods underscore the necessity for ongoing research and the integration of multifaceted therapeutic strategies to effectively address the complexities of Parkinson’s disease [9].

The objective of this review is to systematically map the current evidence regarding the effectiveness of mirror neuron-based rehabilitation techniques in improving motor functions and the overall quality of life in Parkinson’s disease patients, thereby providing a comprehensive overview of their potential integration into standard therapeutic practices.

## 2. Materials and Methods

The present scoping review was conducted following the JBI methodology [24] for scoping reviews. The preferred reporting items for systematic reviews and meta-analyses extension for scoping reviews (PRISMA-ScR) [25] Checklist for reporting was used.

### 2.1. Review Question

We formulated the following research question: “What is the current evidence on the effectiveness of mirror neuron-based rehabilitation techniques in improving motor functions and overall quality of life in patients with Parkinson’s disease, and how can these techniques be integrated into standard therapeutic practices?”.

### 2.2. Eligibility Criteria

Studies were eligible for inclusion if they met the following Population, Concept, and Context (PCC) criteria.

**Population (P):** Individuals diagnosed with Parkinson’s disease, aged over 18 years, regardless of gender or ethnicity.

**Concept (C):** Rehabilitation approaches utilizing mirror neurons as a means of motor learning and recovery.

**Context (C):** Studies conducted without restrictions on geographical or cultural contexts, published in English.

### 2.3. Exclusion Criteria

Studies that did not meet the specific PCC criteria were excluded.

### 2.4. Search Strategy

An initial limited search of MEDLINE was performed through the PubMed interface to identify articles on the topic and then the index terms used to describe the articles were used to develop a comprehensive search strategy for MEDLINE. The search strategy, which included all the identified keywords and index terms, was adapted for use in Cochrane Central, Scopus, PEDro, and Web Of Science. In addition, gray literature and reference lists of all the relevant studies were also searched. The searches were conducted on 30 June 2024 with no date limitation.

**PubMed**: (“Parkinson Disease” [Mesh] OR “parkinson*disease*”) AND (“Mirror Movement Therapy” [Mesh] OR “action observation” OR “motor imagery”).

**Cochrane Library**: (“Parkinson Disease” [Mesh] OR “parkinson*disease*”) AND (“Mirror Movement Therapy” [Mesh] OR “action observation” OR “motor imagery”).

**PEDro**: (“Parkinson Disease”) AND (“action observation” OR “motor imagery” OR “mirror therapy”).

**Web of Science**: (“Parkinson Disease” AND “Mirror Movement Therapy”) OR (“Parkinson Disease” AND “action observation”) OR (“Parkinson Disease” AND “motor imagery”).

**Scopus**: (“Parkinson Disease” AND “Mirror Movement Therapy”) OR (“Parkinson Disease” AND “action observation”) OR (“Parkinson Disease” AND “motor imagery”).

### 2.5. Study Selection

The process described involves a systematic approach to selecting studies for a scoping review. Initially, the search results were collected and refined using Zotero, with duplicates removed. The screening involved two levels: title and abstract review, followed by full-text assessment, both conducted independently by two authors with discrepancies resolved by a third. The selection adhered to the PRISMA 2020 guidelines, ensuring transparency and reliability. This rigorous methodology aimed to identify relevant articles that directly address the research question, maintaining a comprehensive and systematic approach in the review process.

### 2.6. Data Extraction and Data Synthesis

Data extraction for the scoping review was performed using a form based on the JBI tool, capturing crucial details like authorship, publication country and year, study design, patient characteristics, outcomes, interventions, procedures, and other relevant data. The descriptive analyses of these data were conducted, with the results presented numerically to show study distribution. The review process was clearly mapped for transparency, and the data were summarized in tables for the easy comparison and understanding of the studies’ key aspects and findings.

## 3. Results

**Figure 1:**
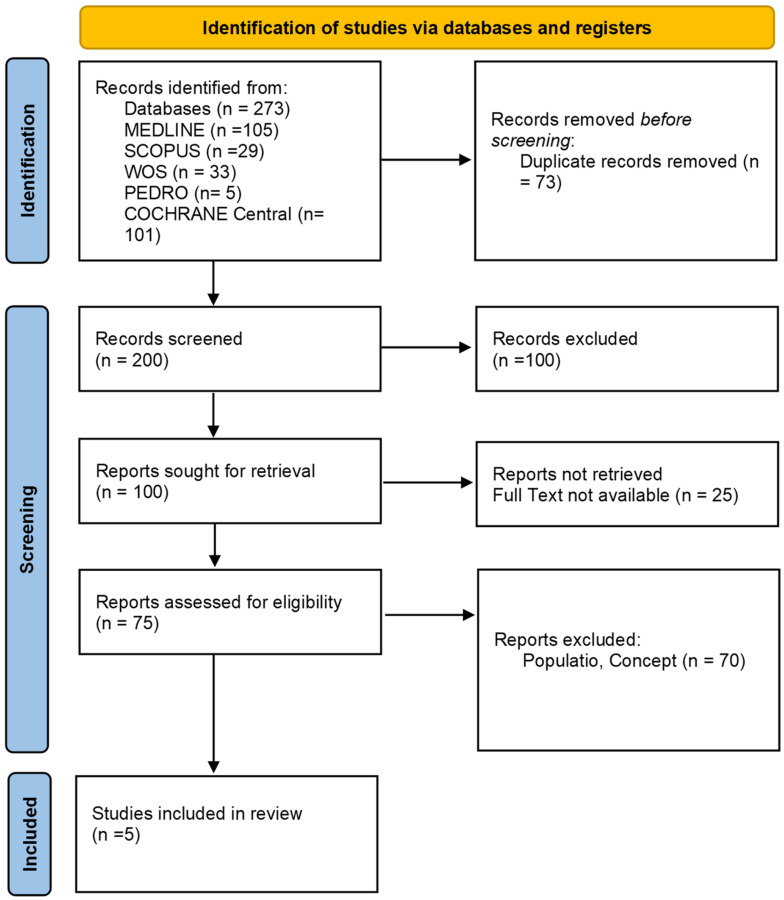
Preferred reporting items for systematic reviews and meta-analyses 2020 (PRISMA) flow diagram.

As presented in the PRISMA 2020 flow diagram (Figure 1), from 273 records identified by the initial literature searches, 268 were excluded and 5 articles were included (Table 1). The quality of the studies was assessed with the PEDro scale (Table 2), AMSTAR 2, and ROB2 (Table 2).

Silvia Lahuerta-Martín and colleagues conducted a systematic review to evaluate the effectiveness of AO- and MI-based therapies in treating PD patients. Their review included six studies with a total of 156 participants. The experimental groups were shown videos with strategies to avoid freezing during walking, and then made to walk, while the control groups were shown videos of landscapes, and then made to walk. The outcomes measured were disease severity, quality of life (QOL), balance, and gait. The results indicated that AO therapy led to a reduction in disease severity and improvements in QOL, balance, and gait. The combination of AO with MI and dual-task exercises proved to be the most effective intervention [26].

Ioannis Giannakopoulos and colleagues conducted a systematic review focusing on the effectiveness of AO-based therapies in PD patients, particularly regarding the improvement of freezing phenomena and gait. This review included seven studies with a total of 194 participants. The experimental groups were shown videos with strategies to avoid freezing episodes during walking or movements with auditory cues or functional movements, and then made to walk, while the control groups were shown videos of landscapes or given auditory cues, and then made to walk. The outcomes measured were disease severity, QOL, balance, freezing, and gait. The findings suggested that AO improved motor control and clinical aspects. However, the amount and frequency of training and the characteristics of the visual stimulus played important roles in the effectiveness of interventions [27].

Elisa Pelosin and colleagues investigated the effects of AO combined with physiotherapy on mobility and FoG in groups of PD patients compared to classical physiotherapy without AO. Their study included 64 subjects with a Hoehn and Yahr scale of 2–3 and a Mini-Mental State Examination score >25. The experimental group (32 subjects) was shown videos with strategies to avoid freezing during walking, and then made to walk, while the control group (32 subjects) was shown videos of landscapes, and then made to walk. The outcomes measured were freezing (FoG-Q), balance (BBS), and gait (TUG, 10 M-WT). The results demonstrated that AO was effective in reducing gait freezing and improving balance and gait. AO was found to be a safe and feasible treatment, representing an additional strategy for physiotherapists [28].

Elisabetta Sarasso and colleagues evaluated the effects of AO and MI on mobility, balance, and brain reorganization after six weeks in PD patients with postural instability and gait disturbances. This study included 25 subjects with a Hoehn and Yahr scale ≤4 and a Mini-Mental Status Examination (MMSE) score ≥24. The experimental group (13 subjects) performed dual-task exercises (balance and gait) combined with AO and MI therapy (DUAL-TASK + AO-MI), while the control group (12 subjects) performed dual-task exercises (balance and gait) alone (DUAL-TASK). The outcomes measured were functional movements, balance (TUG and ABC scale), and gait (10 MWT). The study found that both dual-task training and dual-task combined with AO and MI led to improvements in clinical health and brain reorganization. The most notable result was the increase in rotation speed during dual-task exercises in the group receiving AO and MI, along with greater improvements in balance, QOL, and reduction in gait freezing (FOG) [20].

In contrast, a study by Paula T. Bezerra and colleagues did not find significant improvements in balance, gait, and freezing of gait parameters in PD patients receiving combined AO and MI therapy compared to the control group. This randomized controlled trial included 39 subjects with a Hoehn and Yahr scale of 1.5–3, no dementia, and a Mini-Mental State Examination score ≥18 for illiterate and ≥24 for those with school education. The experimental group (21 subjects) was shown videos of gait training in a kinesthetic modality and gait training (AO and MI), while the control group (18 subjects) was shown educational videos related to Parkinson’s disease and gait training, and then made to walk. The outcomes measured were freezing (FOG-Q), balance (mini BESTest), and gait. The results indicated that the experimental group did not show significant improvements in balance, gait, and freezing parameters compared to the control group [29]. In addition to using international scales and scores, several studies included quantitative analyses for gait. Pelosin et al. [28] utilized a motion capture system to measure specific gait parameters such as stride length, walking speed, gait variability, step time, and double support time. Sarasso et al. [20] employed wearable inertial sensors to analyze gait dynamics, including step count, stride length, gait symmetry, cadence, acceleration, and rotation speed. Giannakopoulos et al. [27] used pressure-sensitive walkways to assess various quantitative gait parameters, such as ground reaction forces, temporal/spatial characteristics, foot pressure distribution, and gait cycle duration. These quantitative analyses provided deeper insights into the specific improvements in gait mechanics and dynamics resulting from the interventions.

## 4. Discussion

The objective of this review is to systematically map the literature on physiotherapeutic treatments that utilize mirror neurons for motor learning in patients with Parkinson’s disease. The findings from the reviewed studies, although varied in their methodologies and objectives, indicate that therapies involving action observation (AO) and motor imagery (MI) can lead to improvements in several functional domains, including freezing reduction, balance, gait, and quality of life (QOL) in both the short and long term. The study by Silvia Lahuerta-Martín et al. [26], which included 156 patients across six studies, demonstrated a significant overall improvement in patients subjected to AO and MI. The analysis highlighted a direct correlation between these rehabilitative techniques and enhanced motor functions, QOL, gait, and balance in the experimental group (EG). Similarly, Ioannis Giannakopoulos et al. [27] found that AO and MI techniques led to improvements in motor functions, including QOL, gait, and balance, in the EG compared to the control group (CG). Elisa Pelosin et al. [28] aimed to observe both short-term and long-term (4-week follow-up) improvements in patients subjected to AO and MI, compared to a control group that received the same functional therapy but was shown static images of non-anthropized landscapes. Conducted on 64 patients over five weeks, with two 45 min sessions per week, this study found that the experimental group maintained improvements in FoG, balance, and gait even after the four-week follow-up. In contrast, the control group exhibited a regression in performance, returning to pre-intervention levels. Elisabetta Sarasso et al. [20] demonstrated that dual-task training combined with AO and MI improved patient mobility for up to two months post-treatment. This study, conducted on 25 patients over six weeks, found that dual-task training with AO and MI led to greater improvements in cognitive walking speed and rotational speed compared to dual-task training alone. Conversely, the study by Paula T. Bezerra et al. [29], conducted on 39 patients over four weeks, did not find a significant correlation between AO and MI therapies and improvements in freezing, balance, and gait. These findings are consistent with earlier studies by Braun et al. and Santiago et al., which also reported similar outcomes. This indicates that the effectiveness of AO and MI may vary across different contexts and patient populations. The findings of this review suggest that while AO and MI therapies hold the potential for improving motor functions and QOL in Parkinson’s disease patients, there is considerable variability in their effectiveness. Across the included RCTs and systematic reviews, action observation (AO) demonstrated more consistent and significant improvements compared to motor imagery (MI) in several outcome measures. Specifically, AO was more effective in improving gait and balance, and reducing disease severity. Studies by Pelosin et al. and Lahuerta-Martín et al. highlighted significant improvements in gait parameters such as walking speed and stride length with AO. Additionally, AO showed better results in enhancing balance, evidenced by superior performance on the Berg Balance Scale (BBS) and Timed Up and Go (TUG) test in studies by Giannakopoulos et al. and Pelosin et al. Quality of life (QOL) also saw marked improvements with AO interventions, as noted in the systematic reviews by Lahuerta-Martín et al. and Giannakopoulos et al. Furthermore, AO contributed to a significant reduction in disease severity scores, as reported in the studies by Lahuerta-Martín et al. [26] and Sarasso et al. [20]. This variability could be attributed to differences in study designs, patient populations, and the specific methodologies employed. AO and MI were frequently integrated into conventional rehabilitation programs across the studies. These programs typically included standard physiotherapy exercises, gait training, and balance training. In addition to AO and MI, several studies utilized complementary therapies such as auditory cues and dual-task training to enhance rehabilitation outcomes. Table 3 below summarizes the rehabilitation programs and complementary therapies used in the included studies.

However, this review has several limitations. The research, selection, data extraction, and analysis were conducted by a single operator without subsequent revisions, introducing a lack of intra-operator and inter-operator reliability. The heterogeneity of the included studies, with variations in sample sizes and disease stages, limits the generalizability of the results and precludes a deeply focused analysis of specific activities. Instead, this review provides a broad, panoramic view of the current state of research. The current literature suggests that AO and MI therapies have the potential to enhance motor functions and QOL in Parkinson’s disease patients. However, further rigorous and standardized research is necessary to fully understand and validate these therapeutic approaches. The variability in study findings underscores the need for more uniform methodologies and larger, more diverse patient populations to better ascertain the efficacy of AO and MI in this context. The outcomes and interventions showed considerable heterogeneity across the included studies. This variability was evident in the specific AO and MI protocols used, the duration and frequency of sessions, and the additional therapies combined with AO and MI, such as gait training, balance training, and dual-task exercises. Moreover, the outcomes measured, including gait, balance, quality of life, disease severity, and cognitive function, were assessed using different scales and tools, further contributing to the heterogeneity. This significant variability could potentially lead to type I errors, where observed differences might be due to random variability rather than the intervention itself. These findings highlight the importance of developing more standardized protocols and outcome measures in future research to reduce heterogeneity and enhance the reliability of the results.

### Clinical Practice Implications

The findings from this review suggest that integrating action observation (AO) and motor imagery (MI) into physiotherapy for Parkinson’s disease patients can potentially improve motor functions [26,30], balance [31], gait [32,33], and quality of life (QOL). These techniques should be considered as complementary therapies alongside conventional treatments, especially for patients experiencing the freezing of gait (FoG) [34,35]. Clinicians should tailor AO and MI interventions to individual patient needs, considering factors such as disease stage and cognitive function. Standardized protocols and further training for therapists on these innovative techniques could enhance their implementation and effectiveness in clinical settings. Regular follow-ups are crucial to assess long-term benefits and adjust treatment plans accordingly.

## 5. Conclusions

This review highlights the potential benefits of integrating action observation (AO) and motor imagery (MI) into physiotherapy for patients with Parkinson’s disease. The studies reviewed suggest improvements in motor functions, balance, gait, and quality of life, although results vary. Despite some promising findings, further rigorous and standardized research is needed to fully validate these therapeutic approaches and optimize their application in clinical practice. The variability in effectiveness underscores the importance of individualized treatment plans and the need for ongoing assessment and adjustment.

## Figures and Tables

**Table 1:** Main characteristics of the included studies. This table summarizes the objectives, participants, interventions, outcomes, and results of various studies on the effectiveness of mirror neuron-based therapies for Parkinson’s disease.

Study	Objective	Participants	Intervention	Outcome	Results	Frequency/Number of Sessions and Follow-Up
Lahuerta-Martín et al., 2022 [26]	Evaluate the effectiveness of AO and MI in PD patients	156 participants in 6 studies	Experimental: shown videos with strategies to avoid freezing during walking; control: shown videos of landscapes	Disease severity, quality of life, balance, and gait	AO therapy reduced disease severity and improved quality of life, balance, and gait. AO combined with MI and dual-task exercises was the most effective.	3 sessions/week for 8 weeks; follow-up at 3 months
Giannakopoulos et al., 2022 [27]	Assess AO-based therapies’ impact on freezing and gait	194 participants in 7 studies	Experimental: shown videos with strategies to avoid freezing or functional movements with auditory cues; control: shown videos of landscapes or auditory cues	Disease severity, quality of life, balance, and gait	AO improved motor control and clinical aspects. Effectiveness influenced by training amount/frequency and visual stimulus characteristics.	2 sessions/week for 10 weeks; follow-up at 6 months
Pelosin et al., 2018 [28]	Investigate AO combined with physiotherapy on mobility	64 participants	Experimental: shown videos with strategies to avoid freezing during walking; control: shown videos of landscapes	Freezing (FoG-Q), balance (BBS), and gait (TUG and 10 M-WT)	AO effective in reducing gait freezing and improving balance and gait. Safe and feasible as an additional physiotherapy strategy.	2 sessions/week for 5 weeks; follow-up at 4 weeks
Sarasso et al., 2021 [20]	Evaluate AO and MI effects on mobility, balance, and the brain	25 participants	Experimental: dual-task exercises with AO and MI; control: dual-task exercises alone	Functional movements, balance, and gait (TUG, 10 MWT, and ABC scale)	Both dual-task and dual-task + AO-MI improved clinical health and brain reorganization. Dual-task + AO-MI group showed greater improvements.	3 sessions/week for 6 weeks; follow-up at 2 months
Bezerra et al., 2022 [29]	Determine AO and MI effects on gait, balance, and freezing	39 participants	Experimental: shown videos of gait training and kinesthetic modality; control: shown educational videos about PD	Freezing (FOG-Q), balance (mini BESTest), and gait	The experimental group did not show significant improvements in balance, gait, and freezing compared to the control.	2 sessions/week for 4 weeks; no follow-up spec

Legend: ABC scale: Activities-specific Balance Confidence scale; AO: action observation; BBS: Berg Balance Scale; FoG: freezing of gait; MI: motor imagery; TUG: Timed Up and Go test; 10 M-WT: 10-Meter Walk Test.

**Table 2:** Quality assessment table using AMSTAR 2 and RoB-2 scales. This table summarizes the quality assessment of the included randomized controlled trials (RCTs) using the PEDro and RoB-2 scales, detailing the methodological rigor and risk of bias.

Study	AMSTAR 2 Scale	RoB-2 Scale	Quality Assessment
**Systematic Reviews**			
Lahuerta-Martín et al., 2022 [26]	High quality	N/A	Comprehensive literature search, the inclusion of high-quality RCTs, proper randomization, adequate follow-up, and clear reporting of findings.
Giannakopoulos et al., 2022 [27]	Moderate quality	N/A	Adequate literature search, the inclusion of RCTs, some issues with blinding, and heterogeneity of the included studies.
**Randomized Controlled Trials**			
Pelosin et al., 2018 [28]	N/A	Low risk of bias	PEDro Scale: 8/10. High methodological quality with random allocation; baseline comparability; the blinding of subjects, therapists, and assessors; adequate follow-up; and intention-to-treat analysis.
Sarasso et al., 2021 [20]	N/A	Low risk of bias	PEDro Scale: 7/10. High methodological quality with random allocation, baseline comparability, the blinding of subjects and assessors, adequate follow-up, and intention-to-treat analysis.
Bezerra et al., 2022 [29]	N/A	Moderate risk of bias	PEDro Scale: 6/10. Moderate methodological quality with random allocation and baseline comparability, but lacking sufficient blinding and intention-to-treat analysis.

Legend: AMSTAR 2: A Measurement Tool to Assess Systematic Reviews 2; PEDro Scale: Physiotherapy Evidence Database Scale; RoB-2 Scale: Revised Cochrane risk-of-bias tool for randomized trials; N/A: Not Applicable.

**Table 3:** Summary of rehabilitation programs and complementary therapies.

Study	Rehabilitation Program	Complementary Therapies Included
Lahuerta-Martín et al., 2022 [26]	Conventional physiotherapy exercises	Gait training and balance training
Giannakopoulos et al., 2022 [27]	Physical therapy focused on motor function improvement	Gait training, auditory cues, and balance training
Pelosin et al., 2018 [28]	Group-based physiotherapy combined with AO	Gait training and balance training
Sarasso et al., 2021 [20]	Dual-task exercises combined with AO and MI	Cognitive tasks during motor exercises
Bezerra et al., 2022 [29]	Gait training in a kinesthetic modality combined with AO and MI	Educational videos about Parkinson’s disease

## Data Availability

No new data were created or analyzed in this study. Data sharing is not applicable to this article.
